# Supplementary material for: Atypical functional connectivity hierarchy in Rolandic epilepsy
Source: Commun Biol. 2023 Jul 10;6:704. doi: 10.1038/s42003-023-05075-8 (PMC10333191; doi:10.1038/s42003-023-05075-8)
Supplement: Supplementary file 2 — Supplementary Information [file 42003_2023_5075_MOESM2_ESM.pdf]

Supplementary Information for

Atypical functional connectivity hierarchy in Rolandic epilepsy

Qirui Zhang[1,2†], Jiao Li [3,4†], Yan He[5], Fang Yang[6], Qiang Xu[2,7], Sara Larivière[8], Boris C. Bernhardt[8], Wei Liao, [3,4], Guangming Lu[1,2*], Zhiqiang Zhang[1,2*]

[†]**These authors contributed equally to this work.**

Correspondence to: Guangming Lu(cjr.luguangming@vip.163.com), and Zhiqiang Zhang (zhangzq2001@126.com)

Author affiliations:

[1] Department of Diagnostic Radiology, Jinling Hospital, the First School of Clinical Medicine, Southern Medical University, Nanjing 210002, China.

[2] Department of Diagnostic Radiology, Jinling Hospital, Nanjing University School of Medicine, Nanjing 210002, China.

[3] The Clinical Hospital of Chengdu Brain Science Institute, School of Life Science and Technology, University of Electronic Science and Technology of China, Chengdu 610054, China.

[4] MOE Key Lab for Neuroinformation, High-Field Magnetic Resonance Brain Imaging Key Laboratory of Sichuan Province, University of Electronic Science and Technology of China, Chengdu 610054, China.

[5] Department of Neurology, Children's Hospital of Nanjing Medical University, Nanjing 210002, China.

[6] Department of Neurology, Jinling Hospital, Nanjing University School of Medicine, Nanjing 210002, China,

[7] College of Automation Engineering, Nanjing University of Aeronautics and Astronautics, Nanjing, 210002, China.

[8] Multimodal Imaging and Connectome Analysis Laboratory, McConnell Brain Imaging Centre, Montreal Neurological Institute and Hospital, McGill University, Montreal, Quebec, H3A 2B4, Canada.

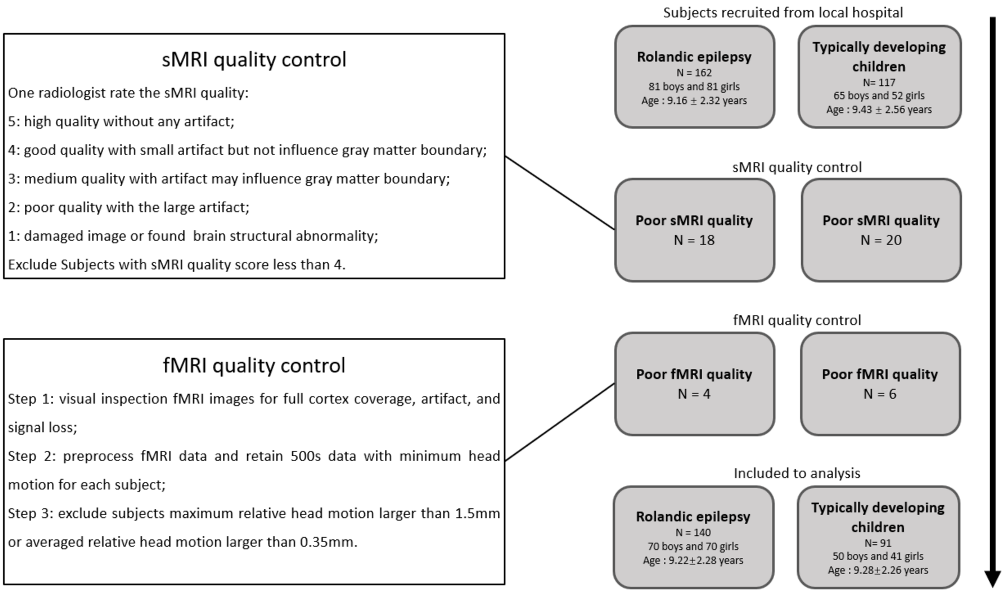

Supplementary Figure 1. Quality control of imaging data

Supplementary Note 1: Age-related changes in each gradient

The first three gradients were showed functional differentiation running from SMN-to-DMN (G1), DMN-to-VN (G2) and SMN-to-VAN (G3) (Supplementary Figure 2a).

We first explored age-related changes in each gradient. Vertex-wise age-related gradient changes showed in Supplementary Figure 2a. Community-wise result found significant positive relationship in DAN(G1), VN(G3), DMN (G2, G3), and DAN(G1), meanwhile negative relationship in VN (G1, G2), DMN(G1), DAN(G3), and SMN(G3), following multiple comparisons correction with a false discovery rate (FDR) procedure (Supplementary Figure 2c).

We found each gradient depicted a different gradual transition across the cortical mantle and the pattern is similar to its age-related changes. Significant spatial correlation was found between mean gradients and its age-related changes (corrected by spin permutation), indicated gradients tended to be disperse with age (Supplementary Figure 2b).

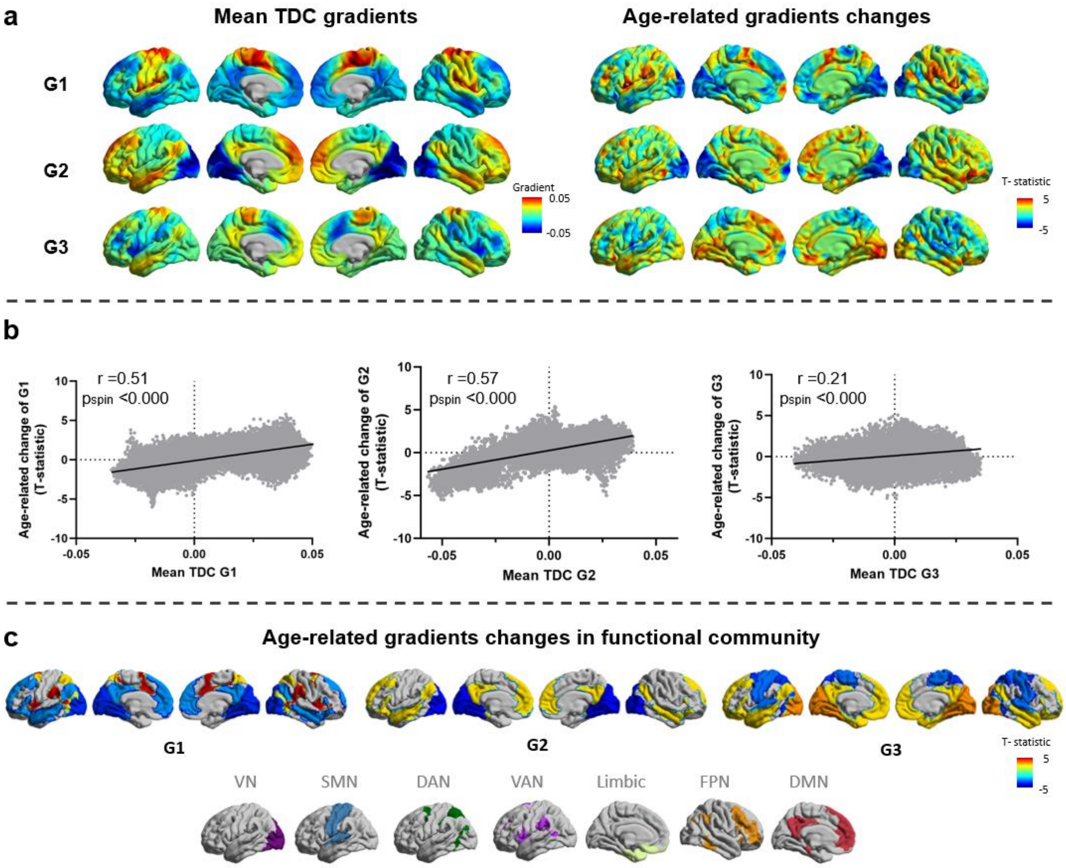

Supplementary Figure 2. Age-related changes of gradients. a Brain surfaces map showed mean TDC gradient values (left) and age-related gradient changes (right). Each gradient depicted a different gradual transition across the cortical mantle and the pattern is similar to its age-related changes. **b** Significant spatial correlation was found between mean gradients and its age-related changes (corrected by spin permutation), indicated gradients tended to be disperse with age. **c** Age-related gradient changes in functional community, following multiple comparisons correction with a false discovery rate (FDR) procedure. Significant positive correlation (warm colored) and negative correlation (cool colored) showed in each gradient. Each network in functional community were labeled in bottom line.

Supplementary Note 2: Age-related changes in gradients eccentricity

Gradients eccentricity showed positive changes in the majority area with age in vertex-wise analysis (Supplementary Figure 3a). The expanded eccentricity with age was also observed in all functional community-wise analyses (false discovery rate (FDR) < 0.05, especially in VN, VAN, FPN, and DMN) and cortical average-wise (TDC and RE group both with $r = 0.32$, $p < 0.01$; Supplementary Figure 3b). In linear regression, we found brain development associated with frontal and occipital lobes positive change in FCD, and with widespread and uniformly negative changes in cortical thickness (Supplementary Figure 3cd). But very similar patterns of eccentricity change after controlling for FCD or cortical thickness (Supplementary Figure 3ef). These results indicate that the gradient eccentricity has an independent predictive effect for age.

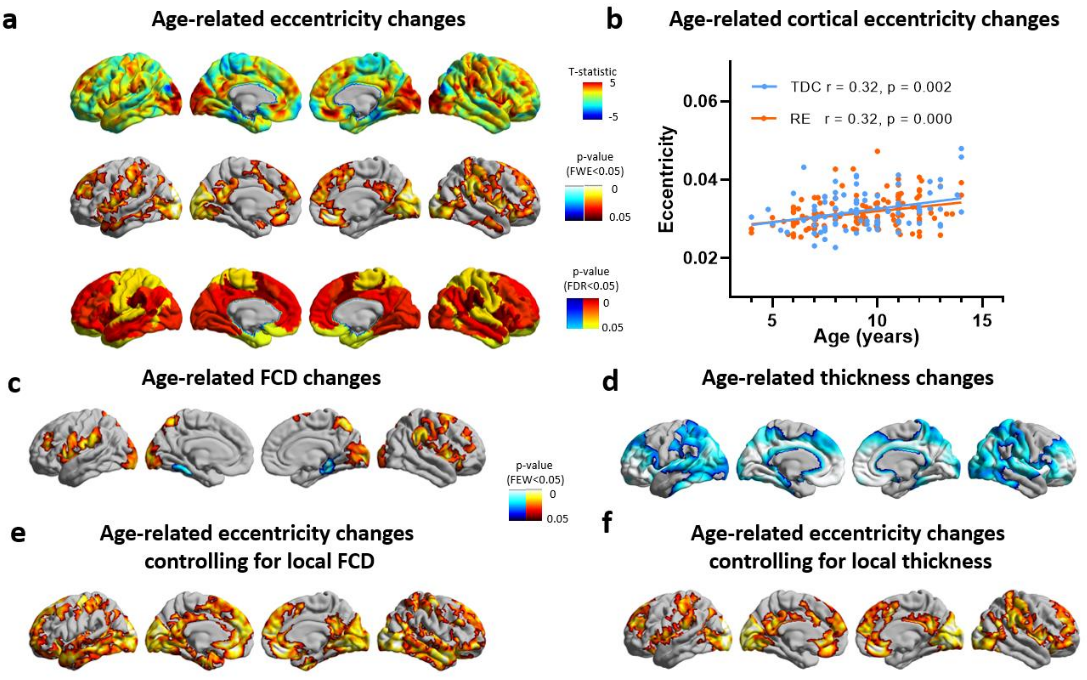

Supplementary Figure 3. Age-related changes in gradients eccentricity. a Age-related eccentricity changes in vertex-wise, network-wise, cortical average-wise (**b**), and significant regions of vertex-wise analysis(**a**) showed on the brain surface. **c** Vertex-wise age-related functional

connectivity density (FCD) changes. **e** Age-related changes to the eccentricity are little influenced by FCD. (D) Age-related thickness changes in vertex-wise. **f** Age-related changes to the eccentricity are little influenced by thickness.

Supplementary Note 3: Sex effect on eccentricity

We also test the eccentricity maturation trend in different sex. We compare eccentricity between RE and TDC using linear models that controlled for effects of age and head motion. We found eccentricity significant higher in female in cortical average-wise analysis (t = 2.47, p = 0.007), especially in the DMN (functional community-wise analysis, false discovery rate qFDR < 0.05) (Supplementary Figure 4ab).

Eccentricity was significant correlated age in cortical average-wise analysis in both male (r = 0.32, p < 0.001) and female (r = 0.31, p < 0.001) (Supplementary Figure 4F). All functional community networks showed significant correlation between eccentricity and age in female, while not significant in SMN and DAN in male (false discovery rate qFDR < 0.05) (Supplementary Figure 4cd). But there was no significant sex × age interaction effect on functional community-wise and cortical average-wise analysis(false discovery rate qFDR < 0.05) (Supplementary Figure 4ef).

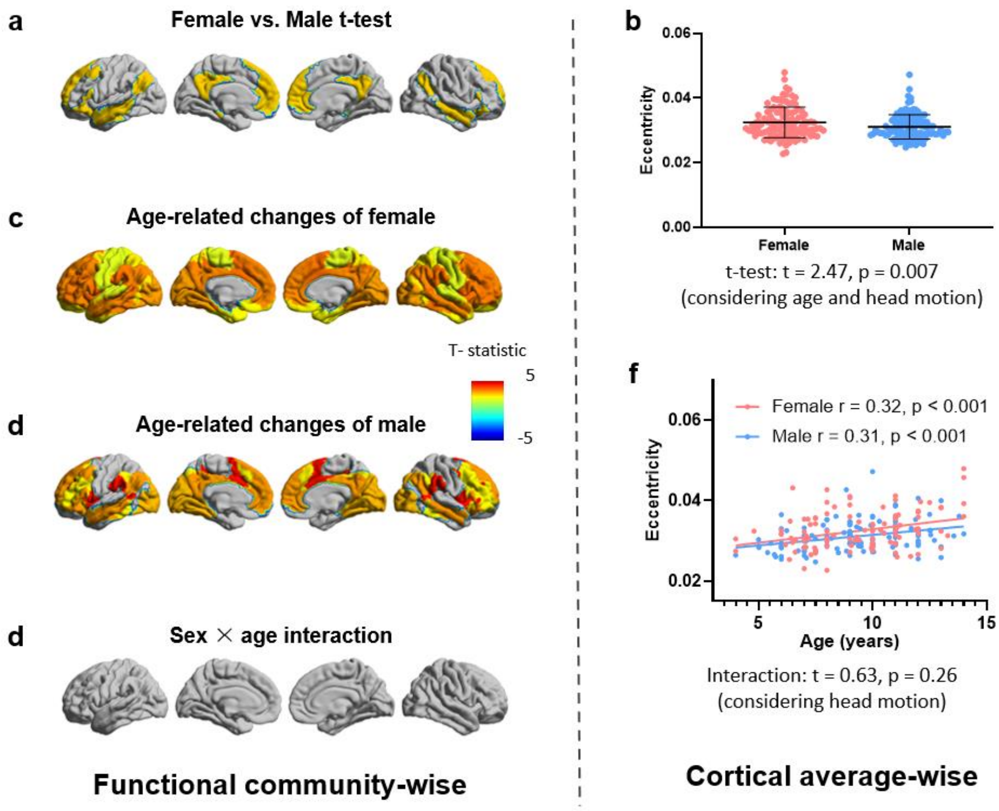

Supplementary Figure 4. Age-related eccentricity changes in female and male. a Functional community-wise t-test between female and male. **b** Cortical average-wise t-test between female and male. **c** Age related eccentricity changes of female. **d** Age related eccentricity changes of male. **e** Functional community-wise sex × age interaction analysis. **f** Cortical average-wise sex × age interaction analysis. Error bars indicate the standard deviation.

Supplementary Note 4: Altered age-related gradient in RE

We used disease × age interaction analysis to observe maturational alteration in each gradient that controlled for effects of sex, head motion. Vertex-wise comparisons (after multiple comparison correction at a family-wise error of $\mathrm{pFWE} < 0.05$) revealed increases and decreases interaction effect of RE.

In G1, RE showed increases interaction effect in occipito-temporal region, fusiform gyrus, together with decreases in middle cingulum, premotor cortex (Supplementary Figure 5a). Functional community analysis found significant interaction effect in VN, which RE showed significant decreases of deceleration (false discovery rate $q\mathrm{FDR} < 0.05$) (Supplementary Figure 5b).

In G2, RE showed increases interaction effect in occipito-temporal region, together with decreases in superior temporal gyrus, hand motor area (Supplementary Figure 5A). Functional community analysis found significant interaction effect in SMN, which RE showed significant abnormal decelerate alone with age (false discovery rate $q\mathrm{FDR} < 0.05$) (Supplementary Figure 5b).

In G3, RE showed increases interaction effect in mouth motor area, superior parietal lobule, occipital lobe, together with decreases in supplementary motor area, Broca’s area (Supplementary Figure 5A). Functional community analysis found significant interaction effect in DAN, which RE showed significant decreases of deceleration (false discovery rate $q\mathrm{FDR} < 0.05$) (Figure6b).

We further case-control compared each gradient between RE and TDC using surface-based linear models that controlled for effects of age, sex, head motion. Vertex-wise comparisons (after multiple comparison correction at a family-wise error of $p\mathrm{FWE} < 0.05$) revealed increases and decreases gradient score in RE.

In G1, RE showed increases gradient in foot motor area, medial prefrontal cortex, anterior Insula, and occipito-temporal region, together with decreases in superior

temporal gyrus, and angular gyrus (Supplementary Figure 6a). Functional community analysis found increases in the DMN network, whereas DAN and VAN showed decreases (false discovery rate *q*FDR < 0.05) (Supplementary Figure 6b).

In G2, RE showed increases gradient in superior temporal gyrus, occipital lobe, together with decreases in posterior cingulum, foot motor area (Supplementary Figure 6A). Functional community analysis found increases in the VN and SMN network, whereas DMN and DAN showed decreases (false discovery rate *q*FDR < 0.05) (Supplementary Figure 6a).

In G3, RE showed increases gradient in foot motor area, occipito-temporal region, orbitofrontal cortex, together with decreases in medial prefrontal cortex, occipito-temporal region, premotor cortex, and superior parietal lobule (Supplementary Figure 6A). Functional community analysis found increases in the VN and VAN network, whereas DMN and DAN showed decreases (false discovery rate *q*FDR < 0.05) (Supplementary Figure 6b).

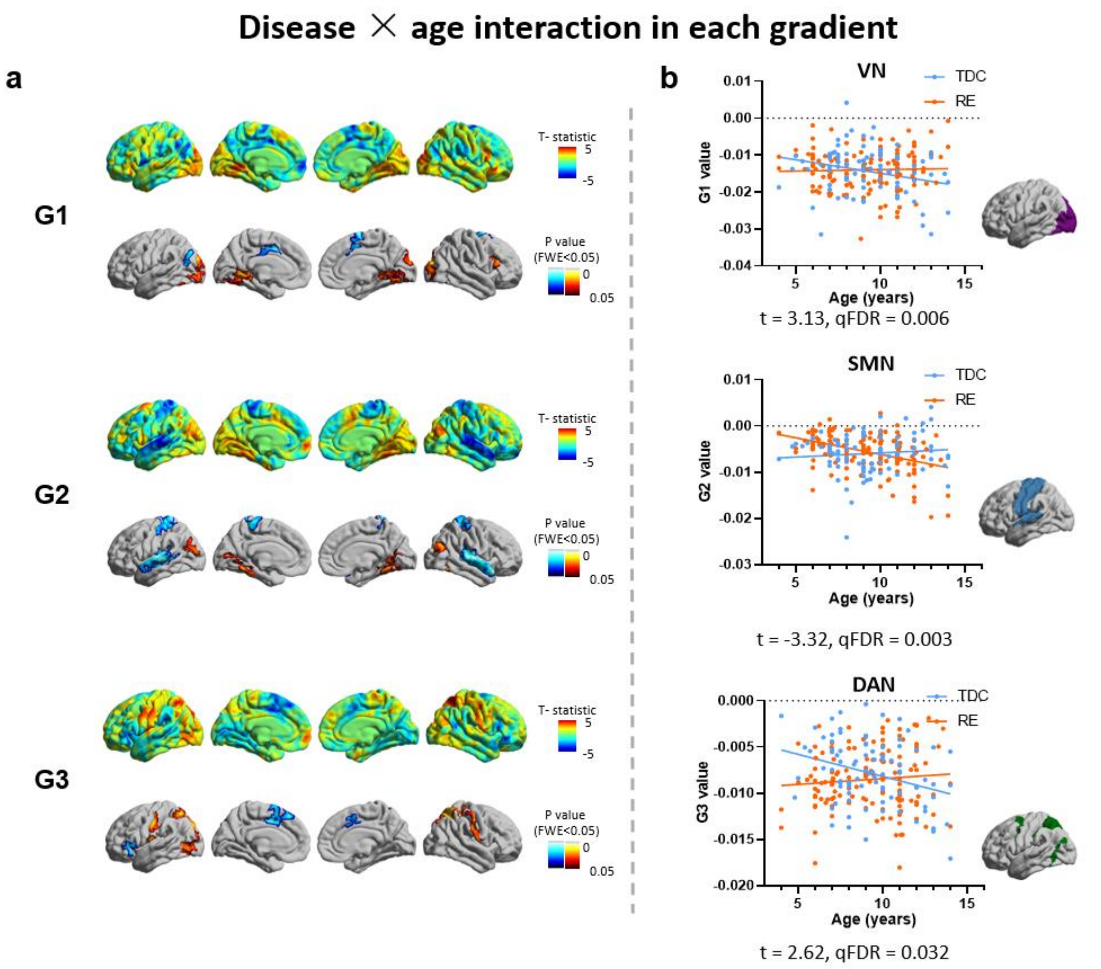

Supplementary Figure 5. Disease × age interaction analysis of each gradient. **a** Surface-based disease × age interaction analysis in each gradient. **b** Community-based disease × age interaction in each gradient.

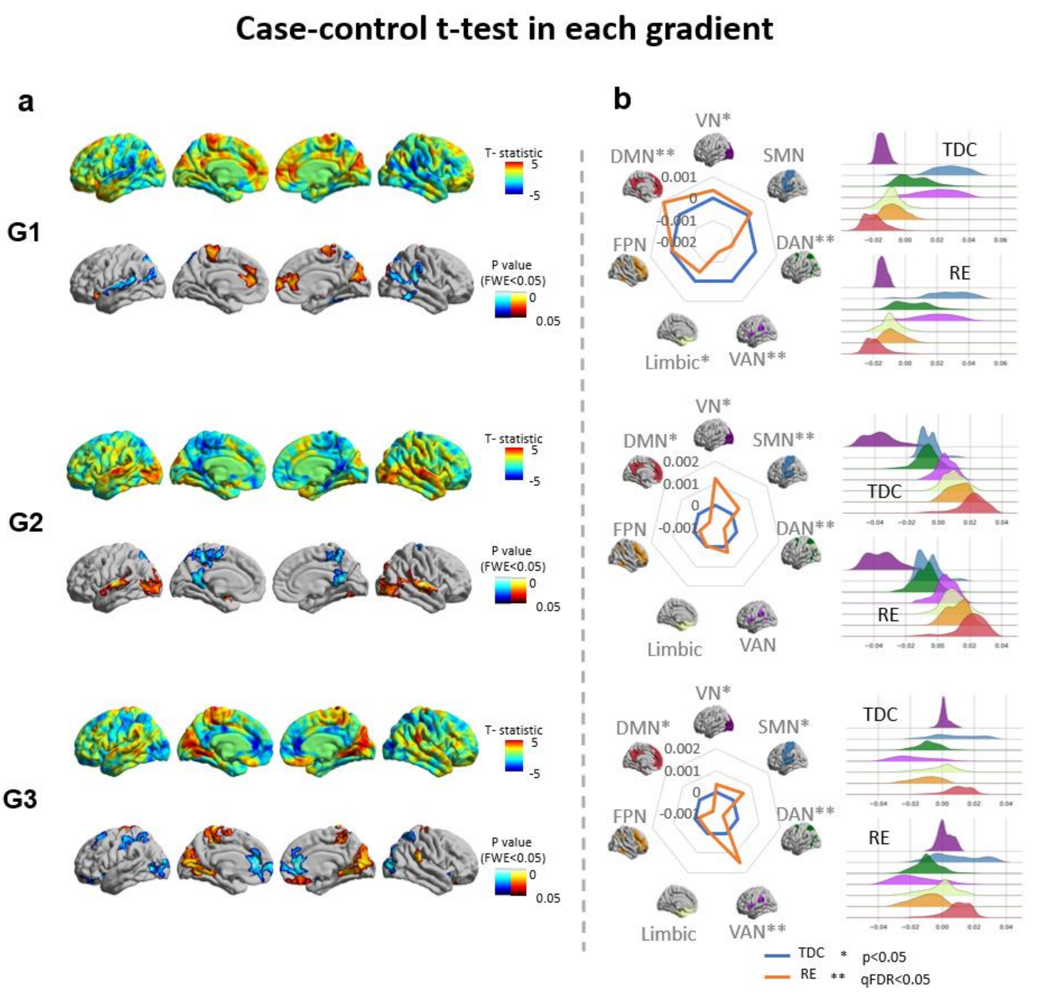

Supplementary Figure 6. Case-control t-test of each gradient between RE and TDC. **a** Surface-based case-control t-statistic comparison between RE and TDC in each gradient. **b** Community-based z-score analysis of gradient score (with respect to TDC) and joy plots in each gradient.

Supplementary Note 5: Reproducibility analysis to disease × age interaction

To determine results stability cross different sample size, we carried out a bootstrap analysis in disease × age interaction tests. We bootstrapped different sample sizes (20%–100% in 20% increments) to calculated disease × age interaction effect. In each sample size 1000 times bootstraps and statistics were performed. Mean z-value of statistics in each sample size showed in Supplementary Figure 7a. The spatial correlations to disease × age interaction calculated by all samples (Supplementary Figure 7b) were showed in Supplementary Figure 7c (all $p_{spin} < 0.0001$, FDR-corrected). Community-wise also calculated, Supplementary Figure 7d showed disease × age interaction z-value in VN cross different sample size.

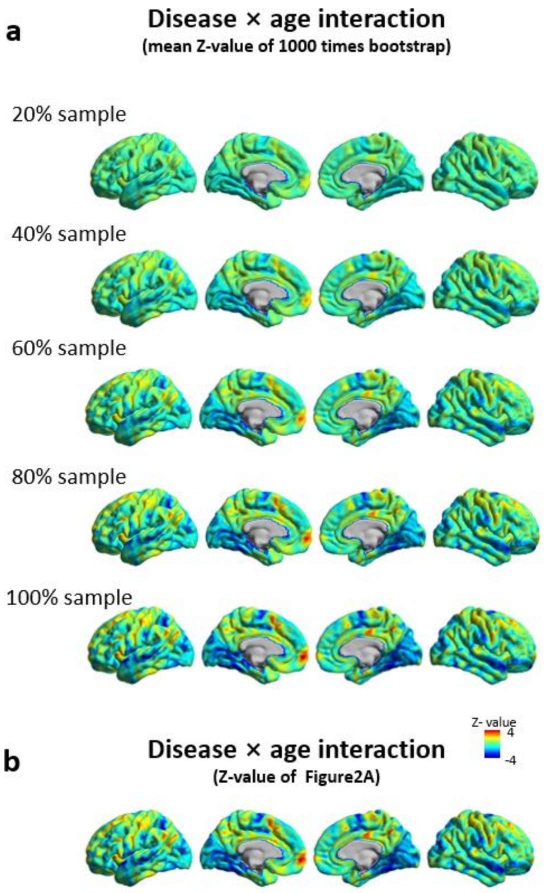

c

Sample size	r (mean Z-value)	r ± std (each Z-value)
20%	0.9936	0.4564 ± 0.0911
40%	0.9988	0.6013 ± 0.0723
60%	0.9991	0.6871 ± 0.0593
80%	0.9994	0.7380 ± 0.0486
100%	0.9995	0.7771 ± 0.0417

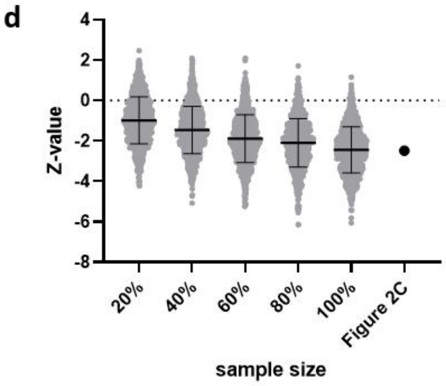

Supplementary Figure 7. bootstrap analysis in disease × age interaction tests. **a**

Mean bootstrap statistical z-value of in each sample size. **b** direct disease × age interaction z-value. **c** The spatial correlations between bootstrap statistical z-value of in each sample size and direct statistical z-value. **d** disease × age interaction z-value in VN cross different sample size. Error bars indicate the standard deviation.

Supplementary Note 6: Antiepileptic drugs (AED) effect on eccentricity in RE

Sixty-three of our patients were treated with AED and 77 were AED-naive. Most of our patients were treated with valproic acid, levetiracetam and oxcarbazepine, with only 2 patients on medications that may affect cognition. The details were as follows: 41 patients were treated with monotherapy (levetiracetam: 21, valproic acid: 14, oxcarbazepine: 7, Lamotrigine: 1), 22 patients were treated with polytherapy (levetiracetam + oxcarbazepine: 9, levetiracetam + valproic acid: 4, levetiracetam + Lamotrigine: 5, valproic acid + Carbamazepine: 1, valproic acid + Phenobarbital: 1, Lamotrigine + Topiramate: 1, oxcarbazepine + Lamotrigine: 1).

We also test AED effect on eccentricity maturation in RE. Functional community-wise and cortical average-wise were enroll to this analysis. We compare eccentricity between AED-medicated and AED-naive patients using linear models that controlled for effects of sex, age, and head motion. We found significant lower eccentricity in DMN and FPN of AED-medicated patients (functional community-wise analysis, false discovery rate qFDR < 0.05) (Supplementary Figure 8a), but did not find significant eccentricity difference in cortical average-wise analysis (t = 1.29, p = 0.09) (Supplementary Figure 8b).

Eccentricity was significant correlated in cortical average-wise analysis in both AED-medicated (r = 0.32, p = 0.010) and AED-naive (r = 0.30, p = 0.006) patients (Supplementary Figure 8f). All functional community networks showed significant correlation between eccentricity and age in AED-naïve patients (Supplementary Figure 8c), while not significant in VN in AED-medicated patients (false discovery rate qFDR < 0.05) (Supplementary Figure 8d). But there was no significant AED × age interaction effect on functional community-wise and cortical average-wise analysis(false discovery rate qFDR < 0.05) (Supplementary Figure 8e).

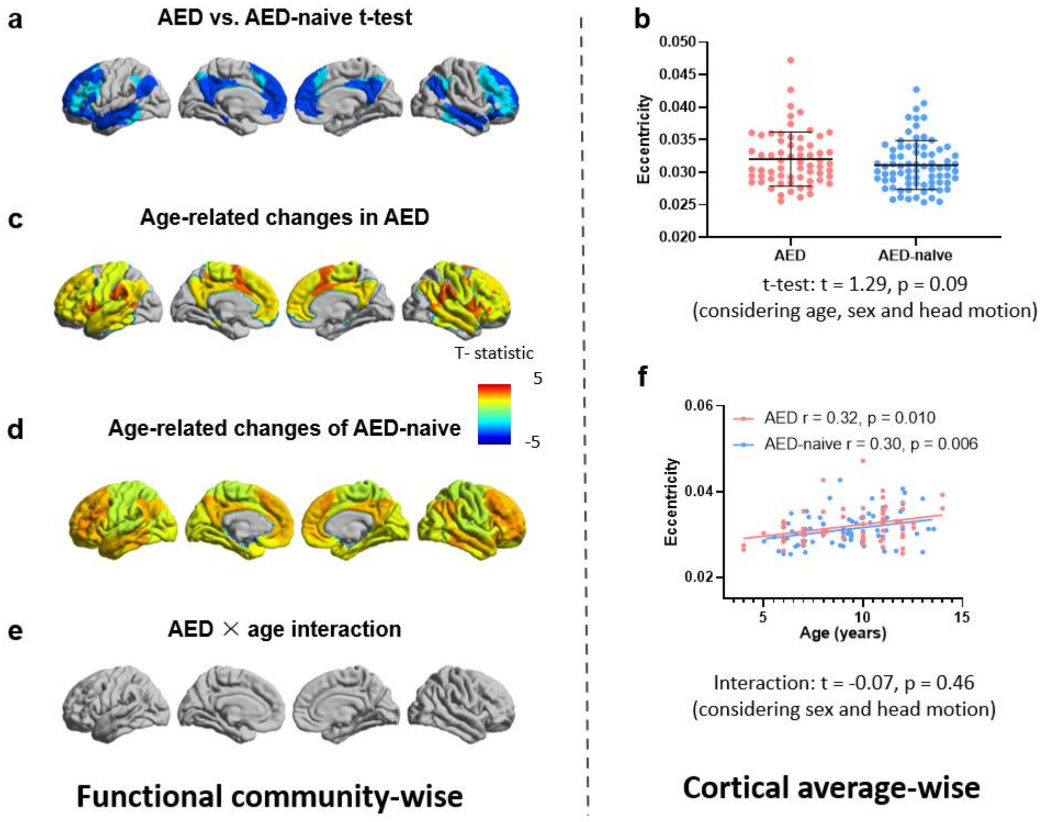

Supplementary Figure 8. AED effect on eccentricity maturation in RE. a Functional community-wise t-test between AED-medicated and AED-naive patients. **b** Cortical average-wise t-test between AED-medicated and AED-naive patients. **c** Age related eccentricity changes of AED-medicated patients. **d** Age related eccentricity changes of AED-naive patients. **e** Functional community-wise AED × age interaction analysis. **f** Cortical average-wise AED × age interaction analysis. Error bars indicate the standard deviation.

Supplementary Note 7: Epileptic discharge effect on eccentricity in RE

A subset of children in our database performed simultaneous EEG-fMRI for some of the patients (n = 48) on a 32 channel MRI-compatible EEG (Brain Product, Munich, Germany, 5k Hz sampling rate) and a 3 T Siemens TimTrio, MRI scanner (Erlangen, Germany) as previous described. Patients were instructed to stay awake and not to fall asleep. Foam pads were used to help secure the EEG leads, minimize motion, and improve patient comfort. For EEG recording, FCz was set as the reference and electrocardiography was recorded using an electrode placed on the back.

EEG data was offline-processed to remove gradient and ballisto-cardiogram artifacts using the Brain Vision Analyzer 2.0 software. Interictal epileptiform discharges were marked on artifact-removed EEG by an experienced electroencephalographer and an epileptologist. Among these patients, 30 had at least one centra-temporal-spike (CTS) discharge.

Among patients who underwent EEG-fMRI, we divided the patients into two subgroups based on the with (N = 30) or without (N = 18) of epileptic discharges during the examination. We compare eccentricity between two subgroups using linear models that controlled for effects of sex, age, and head motion. We found slightly lower eccentricity in patients with epileptic discharges, but not significant on functional community-wise (false discovery rate qFDR < 0.05) and cortical average-wise analysis ($t = 0.95$, $p = 0.17$).
