# Supplementary material for: Atypical functional connectivity hierarchy in Rolandic epilepsy
Source: Commun Biol. 2023 Jul 10;6:704. doi: 10.1038/s42003-023-05075-8 (PMC10333191; doi:10.1038/s42003-023-05075-8)
Supplement: Supplementary file 5 — Reporting Summary [file 42003_2023_5075_MOESM5_ESM.pdf]

nature portfolio

Corresponding author(s): Zhiqiang Zhang; Guangming Lu

Last updated by author(s): Jun 22, 2023

Reporting Summary

Nature Portfolio wishes to improve the reproducibility of the work that we publish. This form provides structure for consistency and transparency in reporting. For further information on Nature Portfolio policies, see our Editorial Policies and the Editorial Policy Checklist.

Statistics

For all statistical analyses, confirm that the following items are present in the figure legend, table legend, main text, or Methods section.

n/a	Confirmed	
☐	☒	The exact sample size (*n*) for each experimental group/condition, given as a discrete number and unit of measurement
☐	☒	A statement on whether measurements were taken from distinct samples or whether the same sample was measured repeatedly
☐	☒	The statistical test(s) used AND whether they are one- or two-sided<br>*Only common tests should be described solely by name; describe more complex techniques in the Methods section.*
☐	☒	A description of all covariates tested
☐	☒	A description of any assumptions or corrections, such as tests of normality and adjustment for multiple comparisons
☐	☒	A full description of the statistical parameters including central tendency (e.g. means) or other basic estimates (e.g. regression coefficient) AND variation (e.g. standard deviation) or associated estimates of uncertainty (e.g. confidence intervals)
☐	☒	For null hypothesis testing, the test statistic (e.g. *F*, *t*, *r*) with confidence intervals, effect sizes, degrees of freedom and *P* value noted<br>*Give P values as exact values whenever suitable.*
☒	☐	For Bayesian analysis, information on the choice of priors and Markov chain Monte Carlo settings
☒	☐	For hierarchical and complex designs, identification of the appropriate level for tests and full reporting of outcomes
☐	☒	Estimates of effect sizes (e.g. Cohen's *d*, Pearson's *r*), indicating how they were calculated

Our web collection on statistics for biologists contains articles on many of the points above.

Software and code

Policy information about availability of computer code

Data collection	All participants underwent functional and anatomical data acquisitions on a 3T MRI scanner (SIEMENS Trio Tim, Siemens Healthcare)
Data analysis	Anatomical (T1-weighted) and functional data were processed using FreeSurfer,<br>Functional connectivity manifolds were generated from preprocessed fMRI data using BrainSpace (https://github.com/MICA-MNI/BrainSpace),<br>Statistical analyses were performed using a vertex linear model implemented in SurfStat (https://math.mcgill.ca/keith/surfstat).

For manuscripts utilizing custom algorithms or software that are central to the research but not yet described in published literature, software must be made available to editors and reviewers. We strongly encourage code deposition in a community repository (e.g. GitHub). See the Nature Portfolio guidelines for submitting code & software for further information.

Data

Policy information about availability of data

All manuscripts must include a data availability statement. This statement should provide the following information, where applicable:

- Accession codes, unique identifiers, or web links for publicly available datasets
- A description of any restrictions on data availability
- For clinical datasets or third party data, please ensure that the statement adheres to our policy

Processed data to reproduce our main findings is available on the Open Science Framework (https://osf.io/48tfa/).

Human research participants

Policy information about studies involving human research participants and Sex and Gender in Research.

Reporting on sex and gender	We used term sex (biological attribute) in this study. Sex was determined from medical records. Sex were considered as a covariant in this study, and sex-based analyses were performed.
Population characteristics	A group of Han nationality patients with Rolandic epilepsy and matched typically developing children were included in this study. They are between the ages of 4 and 14.
Recruitment	The patients were recruited Jinling Hospital. The typically-developing children were recruited from local schools.
Ethics oversight	This research was approved by the medical ethics committee in Jinling Hospital, Nanjing University School of Medicine, and written informed consent was obtained from the guardian of each participating children.

Note that full information on the approval of the study protocol must also be provided in the manuscript.

Field-specific reporting

Please select the one below that is the best fit for your research. If you are not sure, read the appropriate sections before making your selection.

☒ Life sciences ☐ Behavioural & social sciences ☐ Ecological, evolutionary & environmental sciences

For a reference copy of the document with all sections, see nature.com/documents/nr-reporting-summary-flat.pdf

Life sciences study design

All studies must disclose on these points even when the disclosure is negative.

Sample size	This research included 162 cases of Rolandic epilepsy and 117 typically developing children. Sample size was based on availability of data, no statistical methods were used to pre-determine sample size.
Data exclusions	Forty-eight participants were excluded from all analyses because of insufficient quality of the sMRI or fMRI images.
Replication	To determine results stability across different sample sizes, we carried out a bootstrap analysis in disease × age interaction tests of gradient eccentricity.
Randomization	Participants were allocated into patient and control groups based on information on diagnosis. In our neuroimaging analyses, we include sex, age and head motion as covariates.
Blinding	Blinding was not relevant to our study. All group assignments were known based on patient/control status such that neuroimaging analyses could be performed in this study.

Reporting for specific materials, systems and methods

We require information from authors about some types of materials, experimental systems and methods used in many studies. Here, indicate whether each material, system or method listed is relevant to your study. If you are not sure if a list item applies to your research, read the appropriate section before selecting a response.

Materials & experimental systems

n/a	Involved in the study
☒	☐ Antibodies
☒	☐ Eukaryotic cell lines
☒	☐ Palaeontology and archaeology
☒	☐ Animals and other organisms
☒	☐ Clinical data
☒	☐ Dual use research of concern

Methods

n/a	Involved in the study
☒	☐ ChIP-seq
☒	☐ Flow cytometry
☐	☒ MRI-based neuroimaging

Magnetic resonance imaging

Experimental design

Design type: Resting-state and anatomical study.

Design specifications: One scan per participant.

Behavioral performance measures: A part of patients underwent cognitive psychological assessments using Raven's standard progressive matrices (RSPM) and integrated visual and auditory continuous performance test (IVA-CPT).

Acquisition

Imaging type(s): Functional and structural.

Field strength: 3 Tesla.

Sequence & imaging parameters: Functional MRI (fMRI) data were acquired using a single-shot echo-planar imaging sequence (repetition time 2000 ms, echo time 30 ms, field of view 240 × 240 mm2, in-plane matrix 64 × 64, and flip angle 90°). The total scan time for each child was 500 - 2000s, and 30 transverse slices (slice thickness 4 mm, interslice gap 0.4 mm) were acquired, being aligned along the anterior-posterior commissure line. High-resolution 3D T1-weighted anatomical images were acquired using a magnetization-prepared rapid gradient-echo sequence (repetition time 2300 ms, echo time 2.98 ms, flip angle 90, field of view 256 × 256 mm2, and slice thickness 1 mm).

Area of acquisition: Whole brain scan

Diffusion MRI: ☐ Used ☒ Not used

Preprocessing

Preprocessing software: Anatomical (T1-weighted) data were processed using FreeSurfer (v6.0.0, http://surfer.nmr.mgh.harvard.edu). fMRI data for all participants were pre-preprocessed using FreeSurfer with the following steps 25: (1) slice timing correction, (2) rigid body correction for head motion, (3) normalization for global mean signal intensity across runs, (4) bandpass filtering (0.01-0.08 Hz), and (5) nuisance variables (6 motion parameters, white-matter signal, ventricular signal, whole-brain signal, and their temporal derivatives) regression. Following pre-processing, fMRI data were linearly aligned to the corresponding participant's high-resolution anatomical images using boundary-based registration.

Normalization: Functional images were registered to the FreeSurfer cortical surface template (fsaverage6), and down-sampled to fsaverage5 template.

Normalization template: Individual space; fsaverage5.

Noise and artifact removal: Please see above.

Volume censoring: Data not volume censored.

Statistical modeling & inference

Model type and settings: Connectome gradients in children with Rolandic epilepsy were compared to TDC by using vertex-wise linear models. Development alterations were investigated using a disease × age interaction, and disease effect was investigated using case-control t-tests.

Effect(s) tested: Please see above.

Specify type of analysis: ☒ Whole brain ☐ ROI-based ☐ Both

Statistic type for inference (See Eklund et al. 2016): Surface-based whole brain vertex-wise statistic were carry out.

Correction

Surface-based findings were corrected for family-wise errors due to multiple comparisons using a random field theory of pFWE < 0.05.

Models & analysis

n/a	Involved in the study
☐	☒ Functional and/or effective connectivity
☒	☐ Graph analysis
☒	☐ Multivariate modeling or predictive analysis

Functional and/or effective connectivity

Pearson correlation.
